# The genome of the rice planthopper egg parasitoid wasps *Anagrus nilaparvatae* casts light on the chemo- and mechanosensation in parasitism

**DOI:** 10.1186/s12864-022-08656-9

**Published:** 2022-07-28

**Authors:** Ying Ma, Zixiao Guo, Liyang Wang, Bingyang Wang, Tingfa Huang, Bingjie Tang, Guren Zhang, Qiang Zhou

**Affiliations:** 1 State Key Laboratory for Biocontrol, School of Life Sciences, Sun Yat-Sen University, Guangzhou, 510275 China; 2 School of Agriculture, Sun Yat-Sen University, Guangzhou, 510275 China

**Keywords:** *Anagrus nilaparvatae*, Phylogenetic analysis, Olfactory receptor protein, Taste receptor protein, Mechanoreceptor protein

## Abstract

**Background:**

Mymaridae is an ancient insect group and is a basal lineage of the superfamily Chalcidoidea. Species of Mymaridae have great potential for biological control. *Anagrus nilaparvatae*, a representative species of Mymaridae, is ideal for controlling rice planthopper due to its high rate of parasitism and ability to find hosts efficiently in paddy ridges and fields.

**Results:**

Using both PacBio single-molecule real-time and Illumina sequencing, we sequenced and assembled the whole genome of *A. nilaparvatae*, a first for the family Mymaridae. The assembly consists of 394 scaffolds, totaling 488.8 Mb. The assembly is of high continuity and completeness, indicated by the N50 value of 25.4 Mb and 98.2% mapping rate of Benchmarking Universal Single-Copy Orthologs. In total, 16,894 protein-coding genes in the genome were annotated. A phylogenomic tree constructed for *A. nilaparvatae* and other 12 species of Hymenoptera confirmed that the family Mymaridae is sister to all remaining chalcidoids. The divergence time between *A. nilaparvatae* and the other seven Chalcidoidea species was dated at ~ 126.9 Mya. Chemoreceptor and mechanoreceptor genes are important in explaining parasitic behavior. We identified 17 odorant binding proteins, 11 chemosensory proteins, four Niemann-Pick type C2 proteins, 88 olfactory receptors, 12 gustatory receptors, 22 ionotropic receptors and 13 sensory neuron membrane proteins in the genome of *A. nilaparvatae*, which are associated with the chemosensory functions. Strikingly, there is only one pickpocket receptors and nine transient receptor potential genes in the genome that have a mechanosensory function.

**Conclusions:**

We obtained a high-quality genome assembly for *A. nilaparvatae* using PacBio single-molecule real-time sequencing, which provides phylogenomic insights for its evolutionary history. The small numbers of chemo- and mechanosensory genes in *A. nilaparvatae* indicate the species-specific host detection and oviposition behavior of *A. nilaparvatae* might be regulated by relatively simple molecular pathways.

**Supplementary Information:**

The online version contains supplementary material available at 10.1186/s12864-022-08656-9.

## Background

Parasitic wasps are in the spotlight for their potential as biological control agents of crop pests. In the long process of coevolution with their hosts, parasitic wasps have formed unique parasitic behaviors such as oviposition recognition and post-oviposition processing [1]. In the process of parasitism, parasitic wasps recognize host-related chemical information materials through olfaction (smell) and gustation (taste), and the protective traits of hosts through mechanoreception (touch). Facilitated by smell, taste, touch, and other senses, parasitic wasps accurately oviposit in the appropriate internal or external location within their hosts to complete the parasitic behavior [2].

The superfamily Chalcidoidea is a large group, the majority of which are parasitoid wasps. The number of parasitoid wasp species described so far is more than 500,000, accounting for about 75% of the total Hymenoptera species and 10–20% of all described insect species [3]. Mymaridae is an important family in the superfamily Chalcidoidea. Previous diagnostics using phylogenetic analyses, morphological characteristics, molecular data and fossil evidence suggest that Mymaridae is an ancient insect group, which is a basal lineage of Chalcidoidea and sister to all other chalcidoid lineages [4–6]. Fossils of the earliest Chalcidoidea suggest that their closest ancestors might have been small egg parasites [5], a trait retained by nearly all species of Mymaridae.

**Fig. 1:**
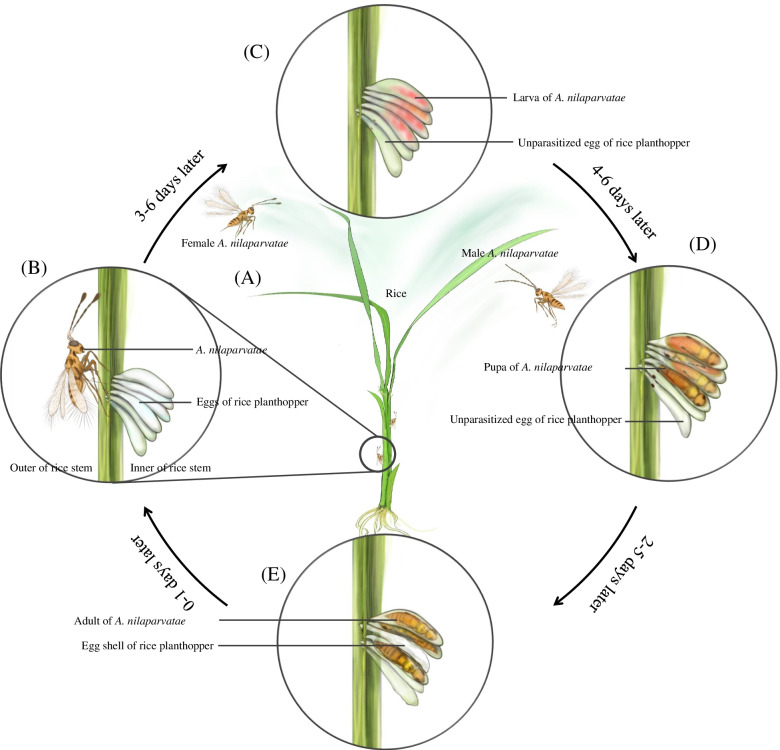
Life Cycle of *Anagrus nilaparvatae*. Wasps were reared under a 14:10 h (L:D) photoperiod at 27 °C,. **A** Females and males *A. nilaparvatae* are attracted to the volatiles of rice or planthoppers and fly to parasitize. **B** Female *A. nilaparvatae* laying eggs. **C** Late larval instars of *A. nilaparvatae* are pink. **D** In the late pupa stage, morphological characters of *A. nilaparvatae* gradually emerge, and the sex of which could be distinguished by antennaes. **E** The adult of *A. nilaparvatae* after emergence bites through the egg shell of rice planthopper and fly out

Rice planthoppers are the most damaging insect pests in rice producing areas of Asia. The mymarid wasp of *Anagrus nilaparvatae* Pang & Wang (Hymenoptera: Mymaridae) is parasitic in the eggs of the planthoppers and is the dominant natural enemy of rice planthoppers. This parasitic behavior makes it valuable of application in rice production [7, 8]. These parasitic wasps spend the winter in paddy ridges, parasitize rice planthoppers in the paddy fields in the spring, and return to the ridges after the rice is harvested (Fig. 1). In the process of finding a host, *A. nilaparvatae* recognize the volatiles released by rice plants that are damaged by rice planthoppers [9–11]. They continuously tap the stem of rice plants to locate host eggs, and they perceive the inner texture of the rice track using the ovipositor to distinguish the host eggs during oviposition (see the supplementary video). It is speculated that olfactory, gustatory and mechanical perception play important roles in the host finding process.

The insect olfactory system consists of several classes of proteins functioning in different steps, including odorant binding proteins (OBPs), chemosensory proteins (CSPs), Niemann-Pick type C2 proteins (NPC2s), olfactory receptors (ORs), ionotropic receptors (IRs), sensory neuron membrane proteins (SNMPs), and odorant-degrading enzymes (ODEs) [12]. In contrast, taste is mediated by gustatory receptors (GRs) [13]. Moreover, insect mechanical perception is signaled by pickpocket receptors (PPKs) and transient receptor potential (TRP) channels [14, 15]. To elucidate the origin of mymarid parasitic wasps, and the genomic basis of their parasitic behavior, we sequenced and assembled the whole genome of *A. nilaparvatae*, the first genome reported in the family Mymaridae. The *A. nilaparvatae* genome assembly provides novel insight for the phylogeny of Chalcidoidea and enables reconstruction of the evolutionary history of gene families related to chemo- and mechanosensation.

## Results

### Genomic assembly information

Before the single-molecule real-time (SMRT) sequencing, we firstly used the Illumina platform to obtain a total of 33.8 Gb of Illumina short reads, with an average sequencing depth of 69X (Table S1). These short reads were used to perform a genome survey. At a K-mer value of 19, we estimated the genome size at 479.2 Mb (Fig. S1).

A total of 368.5 Gb of subreads were obtained from the PacBio SMRT sequencing, with an average coverage of 754X (Table S2). We called 24.6 Gb of CCS (circular consensus sequence) reads from the subreads (Table S2). The Illumina short reads obtained above were used for error correction. The final assembly is 488.8 Mb and consisted of 394 scaffolds. The largest scaffold is 70.7 Mb and the N50 size is 25.4 Mb (Table 1). The genome-wide average GC content is 27.52% and the GC contents of each scaffold are between 20 and 40%.

**Table 1:** Summary of the genome assembly of *A. nilaparvatae*

Statistics	*Anagrus nilaparvatae*
Number of scaffolds	394
Total length	488,841,863 bp
Longest scaffold	74,101,551 bp
Scaffold N50	25,368,259 bp
Scaffold N90	2,930,360 bp
GC content (%)	27.52
N content (%)	0
Gene number	16,861
Average gene length	8076.1 bp
Exon number per gene	5.4
Average exon length	276.0 bp
Excon GC content (%)	32.53

Scaffold N50 (N90) indicates the scaffold size which accumulates to 50% (90%) of the whole genome by ranking all scaffolds from large to small

The genome assembly of *A. nilaparvatae* is of high completeness, which is supported by three assessments. First, 98.2% of the 1367 core BUSCOs (Benchmarking Universal Sing-Copy Orthologs) of insects were completely mapped to the assembly (Table S3). Second, 93.26% of the Illumina clean reads were successfully mapped back to the assembly. Third, 98.83% of the reads of RNA sequencing provided by Ma et al. were successfully mapped to the assembly [16].

### Genome annotation

Before annotation, we masked all the repetitive sequences in the assembly, except sequences of low complexity (0.51%) and simple repeats (1.64%). In total, 55.73% of the genome were repetitive sequences. Particularly, 51.31% of the genome are occupied by interspersed repeats (Table S4). The transposable elements in *A. nilaparvatae* are mostly DNA transposons (36.36% of the genome), long interspersed nuclear elements (LINEs, 7.16%), and long terminal repeat retrotransposons (LTRs, 5.37%) (Table S4; Fig. S2).

Using all of the homology-based, ab initio, and transcriptome-based methods, we predicted 16,861 protein coding genes in the genome of *A. nilaparvatae* (Table S5). The average length of the genes is 8076.1 bp, with an average coding sequence (CDS) length of 1494.8 bp (Figure S3). On average, the genes have 5.4 exons, with the average exon length of 276.0 bp and average intron length of 1490.5 bp (Table 1, Figure S3). Functions of the predicted genes are annotated with the KOG (Eukaryotic Orthologous Groups) database (Figs. S4 and S5).

**Fig. 2:**
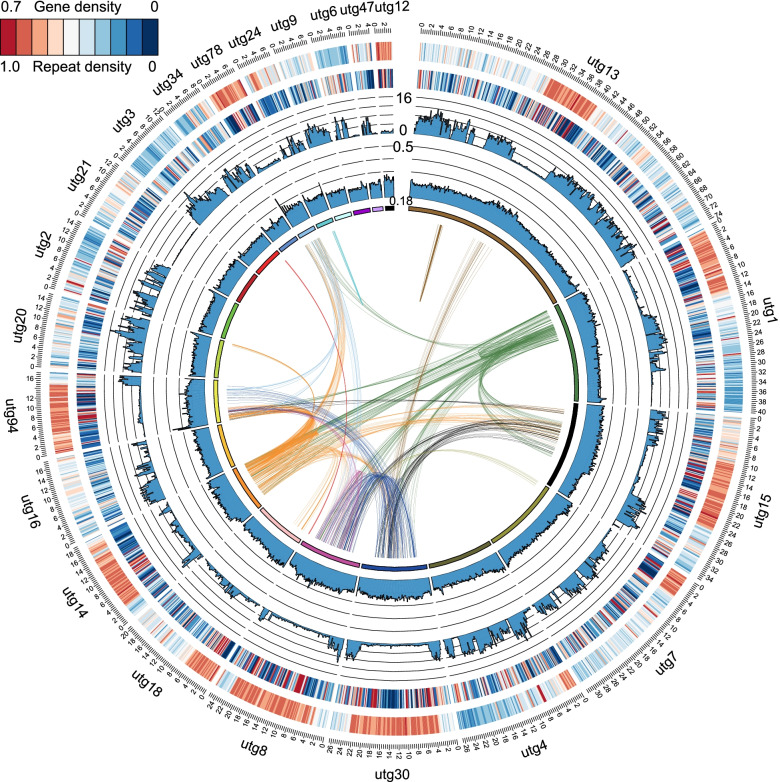
Genome landscape of the parasitoid wasp *Anagrus nilaparvatae*. The letters and numbers outside the circle represent the scaffold label (scaffold length > 3 Mb). From outer to inner circles: heat map of repeat sequence density, heat map of gene density, density of single nucleotide variants, and GC content. The sliding window size is 200 kb. The innermost line shows the collinear genes within the genome, a line connecting a pair of genes

To have a view of the landscape of the genome, the density of repeat sequence, the density of genic sequence, the density of heterozygous site, and GC content were visualized by sliding window of 200 kb (Fig. 2). We also identified 84 genomic blocks with at least five pairs of genes that are in collinearity within the genome (Fig. 2). These genomic blocks covered 897 genes in total.

### Phylogenetic analysis and gene family evolution

**Fig. 3:**
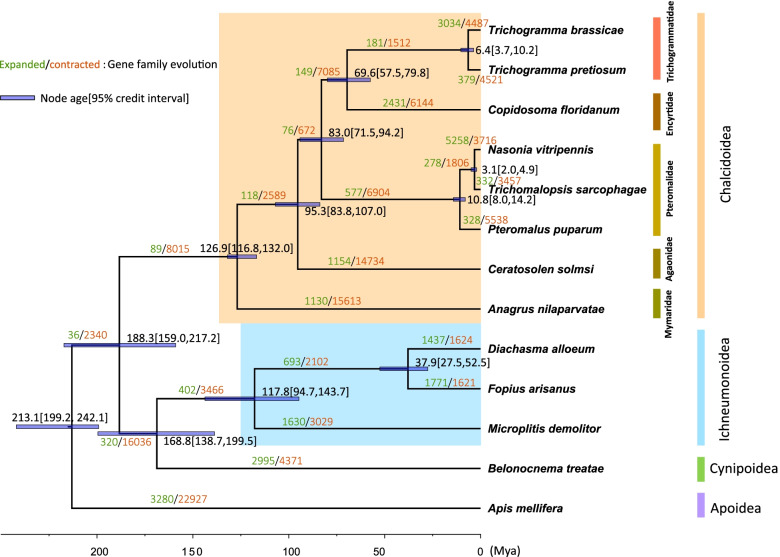
Phylogenomic analyses of the parasitoid wasp *Anagrus nilaparvatae* and 12 related species. The maximum-likelihood phylogenetic tree was constructed for *A. nilaparvatae* and 12 other hymenopterans based on genomewide single-copy orthologs. *Apis mellifera* was used as outgroup. The black numbers on the nodes indicate divergence times (Mya), with error bars indicating 95% credit intervals. The expansion (green) and contraction (red) of gene families are shown on the branches

We used the 205 groups of single-copy orthologs to construct a maximum likelihood tree for *A. nilaparvatae* and 12 other hymenopteran species, with *A. mellifera* specified as the outgroup (Fig. 3). Two main lineages were identified, a monophyletic group of chalcidoids (eight species) and a clade comprising Ichneumonoidea and Cynipoidea. The three species of Ichneumonoidea also formed a monophyletic group. Within Chalcidoidea, *A. nilaparvatae* was the most basal lineage, sister to all the other species of Chalcidoidea. *Ceratosolen solmsi* is second basal, sister to a clade of Trichogrammatidae (*Trichogramma brassicae*, *Trichogramma pretiosum*) *+* Encyrtidae (*Copidosoma floridanum*), and a clade of Pteromalidae (*Nasonia vitripennis*, *Trichomalopsis sarcophagae*, *Pteromalus puparum*). Calibrated by the fossils of known age [17] and previous estimations [5], we dated the split of *A. mellifera* from the others (species of Terebrantia) at about 213.1 million years ago (Mya), with 95% credit interval (CI, credit interval) of 199.2-242.1 Mya. Chalcidoidea diverged from Ichneumonoidea + Cynipoidea at about 188.3 Mya (95% CI: 159.0-217.2), and the divergence between Ichneumonoidea and Cynipoidea is dated at about 117.8 Mya (95% CI: 94.7-143.7). The divergence time estimation between *A. nilaparvatae* and other chalcidoids was about 126.9 Mya (95% CI: 116.8-132.0).

The CAFE (Computational Analysis of gene Family Evolution) analysis showed that gene family contraction overwhelmed expansion in most species except *Fopius arisanus* and *N. vitripennis*. Notably, *A. nilaparvatae* and *C. solmsi* have experienced striking contractions in the number of gene families.

### Chemo- and Mechano-sensory related genes

We identified 17 OBPs, 11 CSPs, 4 NPC2s, 88 ORs, 12 GRs, 23 IRs, 13 SNMPs, 1 PPK, and 9 TRPs in the genome of *A. nilaparvatae*. Detailed information of the numbers identified of the other 12 hymenopteran species can be found in Table 2.

**Table 2:** The number of candidate chemo- and mechano-sensory related genes of *Anagrus nilaparvatae* and other hymenopteran species

statistics	Mymari-dae	Agaoni-dae	Pteromalidae			Encyrti-dae	Trichogrammatidae		Braconidae			Cynipi-dae	Apidae
	*Anil*^*2*^	*Csol*^*2*^	*Ppup*^*1*^	*Tsar*^*2*^	*Nvit*^*1*^	*Cflo*^*2*^	*Tpre*^*2*^	*Tbra*^*3*^	*Dall*^*2*^	*Fari*^*2*^	*Mdem*^*2*^	*Btre*^*1*^	*Amel*^*1*^
Genome size (Mb)	466.2	280.4	338.1	236.4	297.3	53.9	187.64	235.4	384.4	153.6	241.2	1538.7	225.2
Genes (n)	16,861	11,412	17,656	16,084	24,388	12,932	13,395	16,905	13,480	11,691	12,892	14,488	9935
OBP	17	8(7)	57	48	78(90)	27	33	4	16 (15)	12	13	29	19 (21)
CSP	11	8	9	7	10 (9)	10	9	4	9 (9)	10	9	17	9 (6)
NPC2	4	2	6	5	7	2	8	1	4	3	8	3	3
OR	88	46	145	156	333 (301)	67	118	15	216 (201)	218	147 (218)	89	231 (170)
GR	12	5 (5)	10	36	73 (58)	19	26	15	39 (40)	32	25 (85)	23	15 (10)
IR	23	29	35	36	90 (19)	30	44	16	40 (56)	51	35	31	34 (18)
SNMP	13	11	12	9	25	22	29	14	18	17	18	20	24 (8)
PPK	1	4	9	10	17	9	10	6	8	14	5	8	9 (8)
TRP	9	11	12	10	45 (12)	14	36	9	16	23	23	36	45 (13)

The assembly statistics of genomes for other hymenopteran species were obtained from NCBI (https://www.ncbi.nlm.nih.gov/). Gene number refers to the number of candidate genes screened by our blast (e-value 10^-10), and the numbers in brackets were reported in other references [18–29]. Superscripts 1,2 and 3 represent assembly levels of Chromosome, Scaffold and Contig, respectively.

*Anil* A. nilaparvatae, *Amel* Apis mellifera, *Btre* Belonocnema treatae, *Cflo* Copidosoma floridanum, *Csol* Ceratosolen solmsi, *Dall* Diachasma alloeum, *Fari* Fopius arisanus, *Mdem* Microplitis demolitor, *Nvit* Nasonia vitripennis, *Ppup* Pteromalus puparum, *Tbra* Trichogramma brassicae, *Tpre* Trichogramma pretiosum, *Tsar* Trichomalopsis sarcophagae

**Fig. 4:**
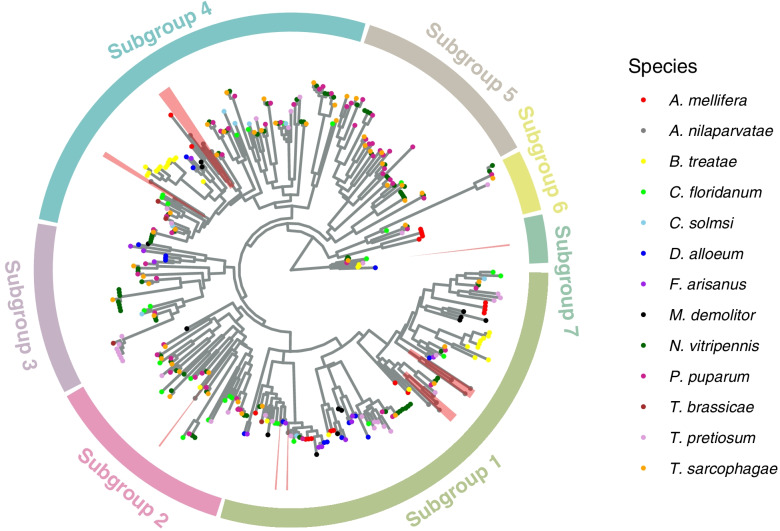
Maximum-likelihood tree of OBPs of the parasitoid wasp *Anagrus nilaparvatae* and other Hymenopteras. Colors of tip nodes indicate species. Genes of *A. nilaparvatae* are highlighted in red shadow

OBPs, CSPs and NPC2s are three kinds of soluble chemoreceptor proteins, which have the function of recognizing, binding and transporting chemical substances such as odor molecular pheromones. These proteins have been found to not only be involved in olfactory sensation, but also play roles in reproduction and anti-stress functions [30–37]. Both CSPs and OBPs have conserved cysteine (Cys) domains, with secondary structures of multiple α-helices [38, 39]. Among the investigated species, the number of OBPs in the three species of Pteromalidae was the largest (48-78 OBPs), followed by the 33 of *T. pretiosum*. The other species have less than 30 OBPs. A phylogenetic analysis of the OBP genes of the 13 Hymenoptera species included in this study were clustered into seven clades, and the 17 OBP genes of *A. nilaparvatae* occurred in four of the seven clades. Particularly, seven of the 17 OBPs of *A. nilaparvatae* are sequentially located on scaffold utg34, suggestive of origination from tandem duplication (Fig. 4). The number of CSP genes of *A. nilaparvatae* (11 members) is only less than that of *Belonocnema treatae* (17 members), and the other species have less than 10 CSPs. The CSPs are phylogenetically clustered into six clades, with *A. nilaparvatae* CSPs occurring in four clades (Fig. S6). In Subgroup2, five CSPs of *A. nilaparvatae* are phylogenetically distant to other members in the same clade, indicating a fast evolutionary rate in *A. nilaparvatae*.

NPC2s were found to be functionally similar to OBPs in recent years [18, 19]. Similar to OBPs and CSPs, NPC2 genes also contain the conserved cysteine domains, but insect NPC2 proteins are mainly composed of β-sheets and form a larger endo-binding cavity [18]. Each of the 13 species studied has fewer than 10 NPC2 candidate genes and four were annotated in the genome of *A. nilaparvatae*. The NPC2s were clustered into three clades (Fig. S7).

The three classes of soluble chemoreceptor proteins mentioned above deliver chemical pheromones or environmental odors to the chemoreceptors of sensory neurons. Subsequently, the three transmembrane transporters, ORs, GRs, and IRs, are responsible for the recognition and discrimination of these signals. Both ORs and GRs belong to the G-protein–coupled receptors (GPCRs), which contain seven transmembrane domains [20, 21]. GRs were initially found to be expressed in the mouth and other taste organs, while ORs were mainly expressed in the antenna [20]. However, GRs were later found to be expressed in olfactory structures, indicating they may function as olfactory receptors [13]. Insect IRs belong to the ionotropic glutamate receptor (IGluR) family, which is a class of conservative ligand-gated ion channels. The structures of IR proteins include extracellular N-terminal (N) and ligand-binding domains (LBD, composed of S1 and S2), three transmembrane domains, ion channel pores and intracellular C-terminals [22]. In recent years, IRs have been found to be involved in taste, temperature and humidity perception in addition to having olfactory functions [23, 24]. SNMPs are a specific class of double transmembrane proteins on olfactory neurons and dendrite membranes that assist ORs in the process of insect sex pheromone recognition [25]. SNMP is a homologous protein of the Mammalian CD36 gene family [26]. The typical SNMP has two transmembrane domains (C-terminal and N-terminal) and an extracellular ring.

Several OR candidate genes were identified in each of the 13 species studied, and the species of Pteromalidae and Braconidae, as well as *Apis mellifera* have over 200 ORs (Fig. S8). We identified 88 ORs in *A. nilaparvatae,* which are likely clustered into six clades. We found less than 40 GRs in each of the 13 species studied, except *N. vitripennis* (73 GRs) (Fig. S9). A total of 12 GR genes were identified in *A. nilaparvatae*. The 13 hymenopteran species generally had 20 to 50 IRs, except the 90 of *N. vitripennis*. The 23 IR genes of *A. nilaparvatae* are dispersed in the seven clades (Fig. S10). Similarly, the numbers of candidate SNMP genes identified in the genomes of the 13 species ranged from 10 to 30, with 13 in *A. nilaparvatae*. The candidate SNMP genes were clustered into 10 subgroups (Fig. S11). The SNMPs of *A. nilaparvatae* occur in all subgroups except Subgroup10. Four SNMPs identified in *A. nilaparvatae* form a clade within the Subgroup1 and are likely the result of two tandem duplication events.

**Fig. 5:**
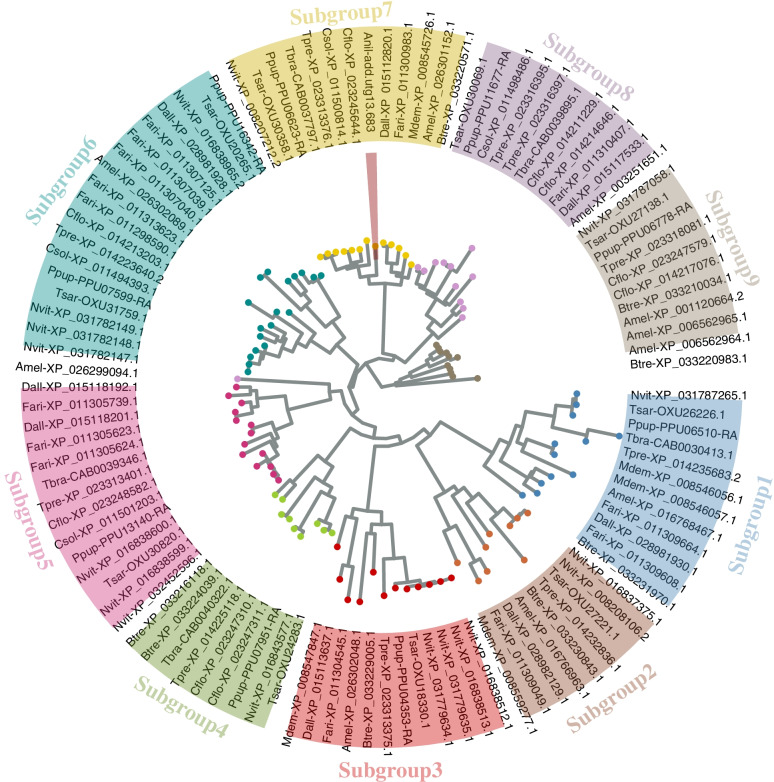
Maximum-likelihood tree of PPKs of *Anagrus nilaparvatae* and other Hymenopteras. The gene of *A. nilaparvatae* is highlighted in red shadow. All gene names are the abbreviation of the species name plus the gene serial number, the gene serial number could be found in NCBI (https://www.ncbi.nlm.nih.gov/) or InsectBase 2.0 (http://v2.insect-genome.com/). Anil, *A. nilaparvatae*; Amel, *Apis mellifera*; Btre, *Belonocnema treatae*; Cflo, *Copidosoma floridanum*; Csol, *Ceratosolen solmsi*; Dall, *Diachasma alloeum*; Fari, *Fopius arisanus*; Mdem, *Microplitis demolitor*; Nvit, *Nasonia vitripennis*; Ppup, *Pteromalus puparum*; Tbra, *Trichogramma brassicae*; Tpre, *Trichogramma pretiosum*; Tsar, *Trichomalopsis sarcophagae*

The insect PPK family is a member of DEG/ENaC (degenerin and epithelial sodium channel), and PPKs usually have a conserved cysteine-rich domain in its extracellular loop and two transmembrane domains. PPKs are involved in mechanosensory functions including water, salt, osmotic potential and pheromones detection [27]. TRP channels in insects are also correlated with mechanosensation [28]. The TRP proteins have six transmembrane helices, the last two of which are located on both sides of the ring that determines ion selectivity. The TRP superfamily plays a key role in the response to photoacoustic chemical temperature and external touch stimuli [14]. We identified less than 10 PPK candidate genes in each of the 13 species studied, except *N. vitripennis* (17) and *F. arisanus* (14) (Table 2). Interestingly, only one PPK gene was found in the genome of *A. nilaparvatae*, which is the lowest among all species. The candidate PPK genes of the 13 hymenopteran species were clustered into nine subgroups, and the only PPK gene of *A. nilaparvatae* is homologous to PPK28 (XP_023245644.4) of *C. floridanum* (Fig. 5). Both *A. mellifera* and *N. vitripennis* have 45 TRPs, and *T. pretiosum* and *B. treatae* have 36. The other species have fewer TRPs. The nine TRPs of *A. nilaparvatae* are dispersed in the five phylogenetic clades of all TRP candidate genes (Fig. S12).

## Discussion

Hymenoptera is the second largest order of insects after Coleoptera. It is estimated that Hymenoptera consists of one million species [29]. However, available genomic resources of hymenopteran species are lacking. At December 2021, the National Center for Biotechnology Information (NCBI) site recorded 228 complete hymenopteran genomes (including 55 Formicidae species), none of which is from a species of the family Mymaridae. Here, we sequenced and assembled the first whole genome of Mymaridae.

Eighty-percent of the sequenced genomes of hymenopteran species are between 180 and 340 Mb in size, with a few exceptions [29]. The size of the *A. nilaparvatae* genome assembly is 488.8 Mb, which is relatively large in Chalcidoidea. This large genome is likely attributed to the high content of repeat sequences (55.73%), which is higher than most chalcidoids. Nonetheless, the content of repeat sequences in the genomes of Hymenoptera can be very low, such as in Apidae (< 10%) [40], or very high, such as in the cynipoid *B. treatae* (80.6%). The large genome of *A. nilaparvatae* contrasts with its extremely small body size (0.6-0.7 mm in body length), suggesting that genome size is not positively correlated with body size. We predicted 16,861 genes in the genome of *A. nilaparvatae*, which is within the range of 12,000-20,000 genes usually found in published Hymenoptera genomes [29]. Notably, the genomes of Hymenoptera species are generally low in GC content, ranging from 30 to 45% [41], but *A. nilaparvatae* reduces the lower bound to 27.52%. Low genome GC content is likely due to GC biased gene transformation and a high recombination rate [42].

Previous studies based on transcriptomes have suggested that Chalcidoidea originated during the late Jurassic (129-81Mya), and the earliest divergence within Chalcidoidea occurred in the early-middle Cretaceous [5, 6]. Our phylogenomic analyses based on whole genomes agrees well with previous assessments that Mymaridae is sister to other groups of Chalcidoidea. Comparing with the previous estimation by Peters et al. [5], we reduced the range of the estimated age of the divergence between Mymaridae and other Chalcidoidea groups from 89 to 208 Mya to 116.8-132.1 Mya. Our estimated mode of 126.9 Mya is well consistent the previous 129.0 Mya. Our phylogeny showed Trichogrammatidae is sister to Encyrtidae, different from the previous suggestion that Trichogrammatidae is a basal lineage (second to Mymaridae) in Chalcidoidea.

The number of chemoreceptor and mechanoreceptor genes differ among species of Hymenoptera, which is related to the complexity of the chemoreceptor and mechanoreceptor genes in these species. Notably, the limitations of available data and methods might have hindered accurate identification of these genes. For example, several previous studies were based on low-quality genome assemblies or transcriptomes [39, 43]. We used the latest high-quality genome assemblies to obtain reliable identification of chemoreceptor and mechanoreceptor genes.

OBPs are the first soluble chemoreceptor protein found in insects. In Hymenoptera, the number of OBP genes varies greatly among species [42, 44]. We found an expansion of the OBP family in Pteromalidae, relative to other hymenopteran species. In addition to Pteromalidae, *T. pretiosum* in Trichogrammatidae also has a large OBP family. The phylogenetic analysis of OBPs showed long branches in Pteromalidae and *T. pretiosum*, suggestive of rapid evolution. The number of CSP genes shows a lower level of differentiation among species. The NPC2 genes are usually found in large numbers in species of Chelicerata, but not in insects [45]. Consistently, we identified less than 10 members of the NPC2 family in each of the 13 hymenopteran species studied. The numbers of OBP, CSP and NPC2 in *A. nilaparvatae* are 17, 11 and 4, respectively, which is within the lower half among the investigated species. Among all species, *C. solmsi* has the lowest number of the three kinds of soluble protein genes, which may be attributed to its strict specificity to its plant host, *Ficus* [46].

Each of the 13 hymenopteran species have a large OR family but a small GR family, both of which belong to the GPCR superfamily [47]. Tandem duplication has been extensively found in the OR family, and the expansion of the OR family is usually accompanied with contraction of the GR family [47]. In addition, the numbers of SNMPs show little difference among Hymenoptera species, and we identified more SNMPs than previous efforts [48].

PPKs play important roles in mechanosensory and other functions. PPKs evolved under the genetic birth-and-death model, which produces lineage-specific expansions that form local clusters in our phylogenetic analyses. Among all insect orders, Hymenoptera has the fewest PPK proteins [49]. We found only one PPK gene in *A. nilaparvatae,* the least in the 13 hymenopterans studied. Similarly, the number of TRP genes in *A. nilaparvatae* is also the smallest of the 13 species. The TRP family is classified into seven subfamilies (TRPC, TRPA, TRPM, TRPML, TRPV, TRPN and TRPP), and TRPC is the “classical TRPs”. The size of the TRP family is generally 13 or 14 in insect species, and the subfamily TRPP is usually absent in Hymenoptera [44]. Our phylogenetic analysis of TRPs identified in the 13 species clustered the candidates into five subgroups, with TRPML and TRPV being not distinguishable.

Among all the 13 species, *N. vitripennis* has the most abundant chemo- and mechanosensory genes, consistent with the observation of more gene family expansions than contractions in the CAFE analysis. *T. pretiosum*, another egg parasitoid, has a large number of PPK and TRP genes, as well as abundant chemoreceptor genes. This result is likely due to the wide range and complex habitats of the host of *T. pretiosum*. In contrast, the chemo- and mechanosensory genes of *A. nilaparvatae* have contracted, with less copies than other hymenopteran species. Interestingly, the host range of *A. nilaparvatae* is very narrow. Similarly, *C. solmsi,* which is strictly coevolving with *Ficus*, also shows a contraction of gene families. This implies a correlation between the numbers of chemoreceptor and mechanoreceptor genes and the single host and simple habitat.

## Conclusions

In this study, we assembled and annotated a high-quality, full-length genome the first time for a species of Mymaridae. The chemosensory and mechanosensory genes of *A. nilaparvatae* and 12 other Hymenoptera species were analyzed. This work provides not only new genomic sequences for the phylogeny of Hymenoptera, but also novel biological insights into host selection and oviposition behavior of egg parasitoids. As the dominant natural enemy of brown planthopper in rice paddy ecosystems, *A. nilaparvatae* is the key biological factor to control this pest. The availability and utilization of the *A. nilaparvatae* genome resources would provide a basis for further protection and utilization of parasitic natural enemies for pest control.

## Materials and methods

### Insects

Individuals of the brown planthopper, *Nilaparvata lugens* (Stål) (Hemiptera: Delphacidae)*,* were collected from rice paddy fields at the farm of the South China Agricultural University (SCAU) in Guangdong Province (N 23°9′3″, E 113°20′2″) in 2016. The collected individuals of brown planthopper were reared with rice hydroponic seedlings. Individuals of *A. nilaparvatae* were collected in the paddy field of the SCAU farm in 2018. The collected individuals of *A. nilaparvatae* were stably cultured for 60 generations on rice seedlings with the eggs of *N. lugens* in an insect cage (120 mesh gauze). The insect cage was placed in an insect incubator (GXZ-380D, Ningbo Jiangnan Instrument Factory, Zhejiang, China), and the rearing conditions were as follows: 14:10 h (L:D) photoperiod, 27 °C temperature, and 80% humidity.

### DNA extraction

The *A. nilaparvatae* wasps used for DNA extraction are F3 descendants of the same pair of ancestors. The third-generation wasps were separately raised in a transparent glass tube (1 mL) to prevent the female wasps from mating with males. After emergence, unmated female *A. nilaparvatae* were selected to produce all male offspring. About 600 male individuals of *A. nilaparvatae* were used for DNA extraction. The wasps were frozen and grounded in liquid nitrogen with a mortar, and we extracted DNA using the Insect DNA Kit (GBCBIO Technologies, Guangzhou, China) according to the manufacturer’s instructions. DNA concentration and purity were examined by Nanodrop 2000c (Thermo Fisher, Waltham, MA, USA).

### Genome assembly and quality control

The genome library was constructed and sequenced in the Tianjin Biochip Corporation (Tianjin, China). A 350 bp (insertion size) pair-end library was constructed by splicing DNA and the library was sequenced on an Illumina HiSeq X ten platform (San Diego, California, USA) according to the manufacturer’s instructions. We used these Illumina reads to estimate the genome size with Jellyfish [50].

We used the PacBio single-molecule real-time technology to sequence the genome. The extracted DNA were sheared and the DNA fragments with lengths of 20-25 Kb were collected using BluePipin (Sage Science, Massachusetts, USA). We constructed a SMRTbell library following the PacBio DNA Template Preparation Kit (PacBio, California, USA). We used 8 M SMRT Cells and V3.0 sequencing reagent to sequence the library on a PacBio Sequel II platform (PacBio, California, USA). We used the HiFiasm software (v0.14) (with the parameters -t32 and -f39) [51] to de novo assemble the genome. The output file containing all primary contigs was used. The Illumina short reads were then aligned to the corrected HiFiasm contigs using BWA-MEM (v0.7.17) [52], and Pilon (v1.2) [53] was used to correct errors in the contigs.

Genomic integrity was assessed by mapping the single-copy homologous genes from the BUSCO database, the raw reads of Illumina sequencing, and the raw reads of RNA sequencing to the genome assembly. The landscape of the assembly was visualized by a circle graph using the Circos software [54]. Meanwhile, based on the mapping of Illumina reads, single nucleotide variants with the genome were identified following the GATK (the Genome Analysis Toolkit) pipeline (https://gatk.broadinstitute.org/hc/en-us).

### Gene annotation

We used RepeatModeler and RepeatMasker (http://www.repeatmasker.org, v4.1.1) to de novo predict and mask repeat sequences in the assembly. With repeats masked, we predicted protein-coding genes using a combination of ab initio, homology-based and transcriptome-based methods. For the homology-based prediction, we used the genomes of four relative species *Apis cerana*, *Bombus vancouverensis*, *Drosophila melanogaster* and *Osmia bicornis* as references, whose genome assemblies and genome annotations are with high quality. We used Exonerate software v2.2.0 [55] to build gene structure based on the homologous alignments. For the ab initio gene prediction, Augustus (V2.5.5) [56] and GeneMark (V4.32) [57] were used. For the transcriptome-based prediction, the full-length transcriptomes of *A. nilaparvatae* [16] were mapped to the genome assembly using Tophat [58]. The transcripts were converted to gene models using Cufflinks v2.2.1 [59]. Finally, EVM (EVidenceModeler) was used to integrate the predicted candidates from different sources, in which the transcriptome-based prediction was given the highest weight [60]. We compared the integrated annotations with the NCBI NR (non-redundant protein sequence) databases and removed the scaffolds where more than half of the genes were non-eukaryotic. We also blasted the final set of predicted genes in the databases of NR, COG (Cluster of Orthologous Groups of Proteins), GO (Gene Ontology) and KEGG (Kyoto Encyclopedia of genes and genomes) to obtain the functional descriptions of genes. We used MCScanX [61] to identify genes in collinearity within the genome.

### Phylogenetic analysis and gene family analyses

OrthoFinder v. 2.3.3 [62] was used to cluster the protein-coding genes of *A. nilaparvatae, C. solmsi, P. puparum, T. sarcophagae, N. vitripennis, C. floridanum, T. pretisum, T. brassicaem,, Diachasma alloeum, F. arisanus, Microplitis demolitor, B. treatae, and A. mellifera* into orthologous groups [63]. We identified 205 single-copy groups (with one member for each species). For each of the single-copy groups, we aligned the protein sequences using MAFFT v7.453 [64] and transformed the alignments to DNA codons using PAL2NAL v. 14.0 [65]. The genes were then concatenated to construct a phylogeny using RAxML v8.2.9 with the maximum likelihood method [66]. *A. mellifera* (Apoidae: Apidae), which belongs to Aculeata, is the outgroup of this evolutionary tree. All the other species are Terebrantia. The phylogenetic tree was dated using the MCMCTREE, which is a part of the software packages PAML v.4.9 [67]. In dating the tree, the root age of the prior was set between 203 and 276 Mya [5]. The divergence between Chalcidoidea and all the other families was constrained at > 130 Mya and the divergence between *A. nilaparvatae* and other Chalcidoidea species was constrained at 99 ~ 130 Mya [5, 16]. We used CAFE v 3.1 to infer the expansion and contraction of gene families along branches of the phylogeny.

To identify OBP, CSP, NPC2, OR, IR, SNMP, GR, PPK and TRP genes that are related to chemo- and mechanosensation, we searched the known sequences of *A. mellifera* and *N. vitripennis* in the genomes of all the 13 species using BLAST V2.7.1 +, following the method commonly used in previous studies [68–70]. We checked the conservative domains of the candidates manually in Pfam (http://pfam.xfam.org/search/sequence) and removed the candidates without typical elements of the domain of the corresponding gene family. The reliable candidates were aligned using MAFFT v7.453 [64]. RAxML V8.2.9 [66] was used to construct gene trees under the optimal substitution model selected by the ProtTest v3.4.2 [71] for each gene family.

## Supplementary Information

**Additional file 1: Table S1.** Statistics of Illumina sequence data. **Table S2.** Statistics of PacBio SMRT sequencing data. **Table S3.** Results of the BUSCO assessment. **Table S4.** Classification of repeat sequences. **Table S5.** Functional annotation of *Anagrus nilaparvatae* genome. **Fig. S1.** Kmer Distribution of Anagrus nilaparvatae genome. **Fig. S2.** Interspersed repeat landscape of the Anagrus nilaparvatae genome. **Fig. S3.** Distributions of the structural characters of the genes predicted in the Anagrus nilaparvatae genome. **Fig. S4.** GO functional classification of the Anagrus nilaparvatae predicted genes. **Fig. S5.** KOG function classification of the predicted genes of Anagrus nilaparvatae. **Fig. S6.** Maximum-likelihood tree of CSPs of Anagrus nilaparvatae and other Hymenopteras. **Fig. S7.** Maximum-likelihood tree of NPC2s of Anagrus nilaparvatae and other Hymenopteras. **Fig. S8.** Maximum-likelihood tree of ORs of Anagrus nilaparvatae and other Hymenopteras. **Fig. S9.** Maximum-likelihood tree of GRs of Anagrus nilaparvatae and other Hymenopteras. **Fig. S10.** Maximum-likelihood tree of IRs of Anagrus nilaparvatae and other Hymenopteras. **Fig. S11.** Maximum-likelihood tree of SNMPs of Anagrus nilaparvatae and other Hymenopteras. **Fig. S12.** Maximum-likelihood tree of TRPs of Anagrus nilaparvatae and other Hymenopteras.

**Additional file 2.**

## Data Availability

Data associated with the *A. nilaparvatae* genome are available in the GenBank/EMBL/DDBJ repository under the accession number PRJNA807850. This Whole Genome Shotgun project has been deposited at DDBJ/ENA/GenBank under the accession JAKSXM000000000. The version described in this paper is version JAKSXM010000000.

## Abbreviations

bp: Base pair
Mb: Million bps
Gb: Giga bps
BUSCO: Benchmarking Universal Single-Copy Orthologs
Mya: Million years ago
OBPs: Odorant binding proteins
CSPs: Chemosensory proteins
NPC2: Niemann-Pick type C2 proteins
ORs: Olfactory receptors
GRs: Gustatory receptors
IRs: Ionotropic receptors
SNMPs: Sensory neuron membrane proteins
PPKs: Pickpocket receptors
TRP: Transient receptor potential
ODEs: Odorant-degrading enzymes
Fig.: Figure
SMRT: Single molecular real time Sequencing
TEs: Transposable elements
LINEs: Long interspersed nuclear elements
LTRs: Long terminal repeat retrotransposons
SINEs: Short interspersed nuclear elements
DNA: DeoxyriboNucleic Acid
RNA: Ribonucleic Acid
Cys: Cysteine
NR: NCBI non-redundant protein sequences
COG: Cluster of Orthologous Groups of Proteins
GO: Gene Ontology
KEGG: Kyoto Encyclopedia of genes and genomes
KOG: Clusters of orthologous groups for eukaryotic complete genomes
Pfam: Protein family
Anil: *Anagrus nilaparvatae*
Amel: *Apis mellifera*
Btre: *Belonocnema treatae*
Cflo: *Copidosoma floridanum*
Csol: *Ceratosolen solmsi*
Dall: *Diachasma alloeum*
Fari: *Fopius arisanus*
Mdem: *Microplitis demolitor*
Nvit: *Nasonia vitripennis*
Ppup: *Pteromalus puparum*
Tbra: *Trichogramma brassicae*
Tpre: *Trichogramma pretiosum*
Tsar: *Trichomalopsis sarcophagae*
USA: The United States of America
CA: California
MA: Massachusetts
