# Supplementary material for: The genome of the rice planthopper egg parasitoid wasps Anagrus nilaparvatae casts light on the chemo- and mechanosensation in parasitism
Source: BMC Genomics. 2022 Jul 28;23:541. doi: 10.1186/s12864-022-08656-9 (PMC9331105; doi:10.1186/s12864-022-08656-9)
Supplement: Supplementary file 1 — Additional file 1: Table S1. Statistics of Illumina sequence data. Table S2. Statistics of PacBio SMRT sequencing data. Table S3. Results of the BUSCO assessment. Table S4. Classification of repeat sequences. Table S5. Functional annotation of Anagrus nilaparvatae genome. Fig. S1. Kmer Distribution of Anagrus nilaparvatae genome. Fig. S2. Interspersed repeat landscape of the Anagrus nilaparvatae genome. Fig. S3. Distributions of the structural characters of the genes predicted in the Anagrus nilaparvatae genome. Fig. S4. GO functional classification of the Anagrus nilaparvatae predicted genes. Fig. S5. KOG function classification of the predicted genes of Anagrus nilaparvatae. Fig. S6. Maximum-likelihood tree of CSPs of Anagrus nilaparvatae and other Hymenopteras. Fig. S7. Maximum-likelihood tree of NPC2s of Anagrus nilaparvatae and other Hymenopteras. Fig. S8. Maximum-likelihood tree of ORs of Anagrus nilaparvatae and other Hymenopteras. Fig. S9. Maximum-likelihood tree of GRs of Anagrus nilaparvatae and other Hymenopteras. Fig. S10. Maximum-likelihood tree of IRs of Anagrus nilaparvatae and other Hymenopteras. Fig. S11. Maximum-likelihood tree of SNMPs of Anagrus nilaparvatae and other Hymenopteras. Fig. S12. Maximum-likelihood tree of TRPs of Anagrus nilaparvatae and other Hymenopteras. [file 12864_2022_8656_MOESM1_ESM.docx]

**Supporting information for**

**The genome of the rice planthopper egg parasitoid wasps *Anagrus nilaparvatae* casts light on the chemo- and mechano-sensing in parasitism**

Ying Ma^1, 2, *^, Zixiao Guo^1, *^, Liyang Wang^1^, Bingyang Wang ^1^, Tingfa Huang ^1^, Bingjie Tang ^1^, Guren Zhang^1^ and Qiang Zhou ^1^

^1^State Key Laboratory for Biocontrol, School of Life Sciences, Sun Yat-Sen University, Guangzhou 510275, China

^2^School of Agriculture, Sun Yat-Sen University, Guangzhou 510275, China

^*^These authors contributed equally to this work.

Correspondence: Qiang Zhou, State Key Laboratory for Biocontrol, School of Life Sciences, Sun Yat-Sen University, [lsszhou@mail.sysu.edu.cn](mailto:lsszhou@mail.sysu.edu.cn)

This file contains:

Supplementary tables S1-S5

Supplementary figures S1-S12

Table S1 Statistics of Illumina sequence data

| Statistics | *Anagrus nilaparvatae* |
| --- | --- |
| Total reads | 112,597,282* 2 |
| Read length | 150 bp |
| PE insert length | 350 bp |
| Total bases | 33,779,184,600 bp |
| Q20 (%) | 98.97% |
| Average coverage | 69X |

Table S2 Statistics of PacBio SMRT sequencing data

| Statistics | *Anagrus nilaparvatae* |
| --- | --- |
| Subreads number | 27,540,646 |
| Total bases of subreads | 368,510,784,709 |
| Average coverage | 754X |
| Circular consensus sequence (CCS) number | 1,646,365 |
| Average CCS length | 14,962 bp |
| Total bases of CCS | 24,632,913,130 bp |

Table S3 Results of the BUSCO assessment

| Statistics | Number | Percentage (%) |
| --- | --- | --- |
| Complete BUSCOs | 1343 | 98.2 |
| Complete and single-copy BUSCOs | 1224 | 89.5 |
| Complete and duplicated BUSCOs | 119 | 8.7 |
| Fragmented BUSCOs | 4 | 0.3 |
| Missing BUSCOs | 20 | 1.5 |
| Total BUSCO groups searched | 1367 | 100 |

Table S4 Classification of repeat sequences

|  | Repeat elements | Number of elements | Length occupied (bp) | Percentage of sequence (%) |
| --- | --- | --- | --- | --- |
| **Retroelements** |  | 128358 | 61669361 | 12.62 |
|  | SINEs | 2273 | 425447 | 0.09 |
|  | Penelope | 17635 | 5896389 | 1.21 |
|  | LINEs | 72703 | 35015629 | 7.16 |
|  | CRE/SLACS | 0 | 0 | 0.00 |
|  | L2/CR1/Rex | 25899 | 11543038 | 2.36 |
|  | R1/LOA/Jockey | 16149 | 13493180 | 2.76 |
|  | R2/R4/NeSL | 685 | 581685 | 0.12 |
|  | RTE/Bov-B | 1092 | 647326 | 0.13 |
|  | L1/CIN4 | 72 | 25066 | 0.01 |
|  | LTR elements | 53382 | 26228285 | 5.37 |
|  | BEL/Pao | 8678 | 6617985 | 1.35 |
|  | Ty1/Copia | 15301 | 5075381 | 1.04 |
|  | Gypsy/DIRS1 | 24908 | 13279068 | 2.72 |
|  | Retroviral | 330 | 103905 | 0.02 |
| **DNA transposons** |  | 678131 | 177766060 | 36.36% |
|  | hobo-Activator | 17410 | 4261704 | 0.87 |
|  | Tc1-IS630-Pogo | 14381 | 3480674 | 0.71 |
|  | En-Spm | 0 | 0 | 0.00 |
|  | MuDR-IS905 | 0 | 0 | 0.00 |
|  | PiggyBac | 1589 | 320910 | 0.07 |
|  | Tourist/Harbinger | 0 | 0 | 0 |
|  | Other (Mirage,P-element, Transib) | 345 | 48429 | 0.01 |
| **Rolling-circles** |  | 26575 | 6683193 | 1.37 |
| **Unclassified** |  | 54795 | 11405206 | 2.33 |
| **Total interspersed repeats** |  |  | 250840627 | 51.31 |
|  |  |  |  |  |
| **Small RNA** |  | 9413 | 3427539 | 0.70 |
| **Simple repeats** |  | 179499 | 7993230 | 1.64 |
| **Low complexity** |  | 49564 | 2476104 | 0.51 |
| **Satellites** |  | 2441 | 1237266 | 0.25 |
|  |  |  |  |  |
| **Bases masked** |  |  | 272437803 | 55.73% |

Table S5 Functional annotation of *Anagrus nilaparvatae* genome.

| Database | Gene number |
| --- | --- |
| Nr | 19088 |
| GO | 12007 |
| KOG | 16894 |
| Kegg | 13079 |
| Swissprot | 12805 |


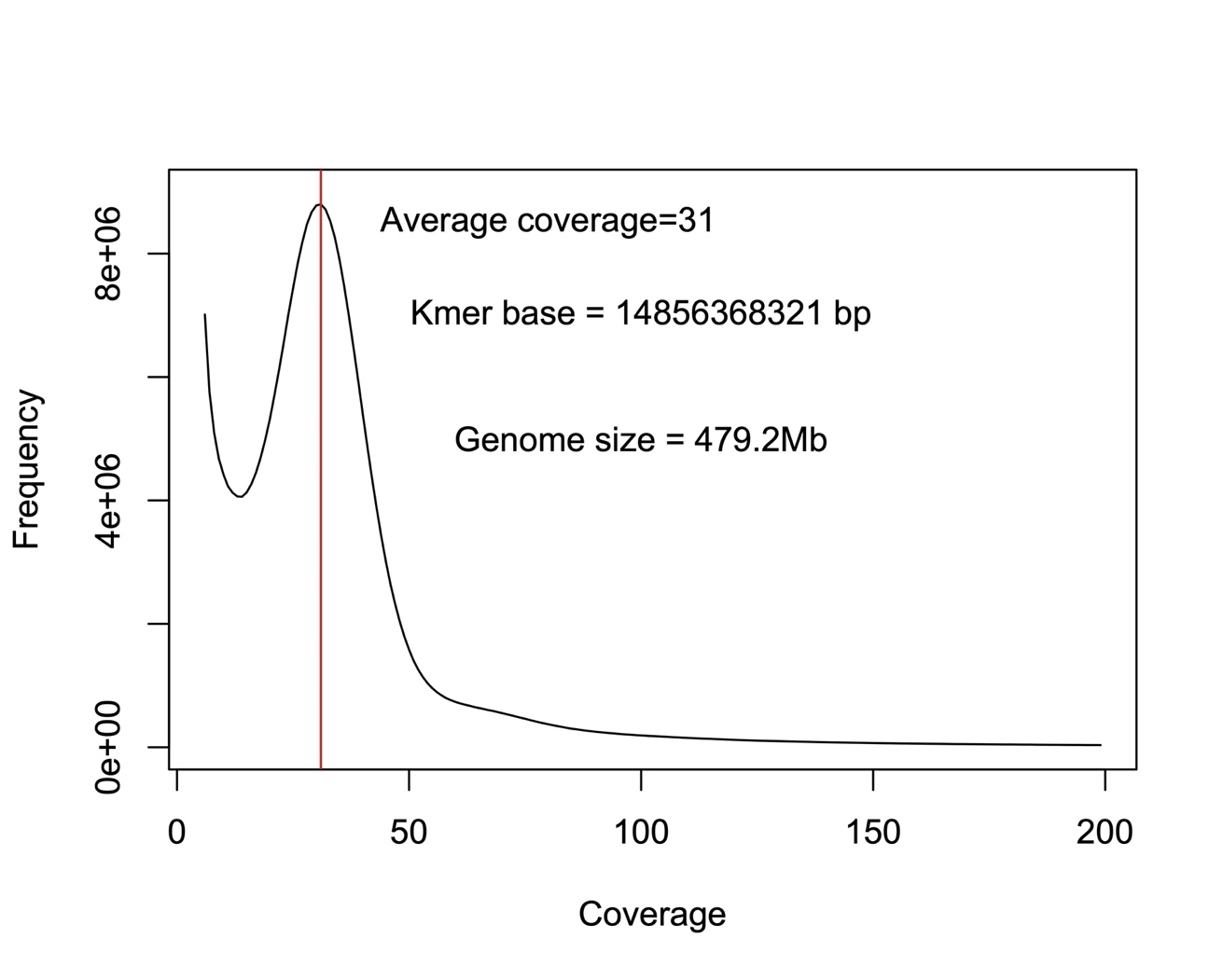


**Fig. S1** **Kmer Distribution of *Anagrus nilaparvatae* genome.** The analysis of the *A. nilaparvatae* genome sequencing data using 19-mer shows that the main peak corresponds to a depth of 31.


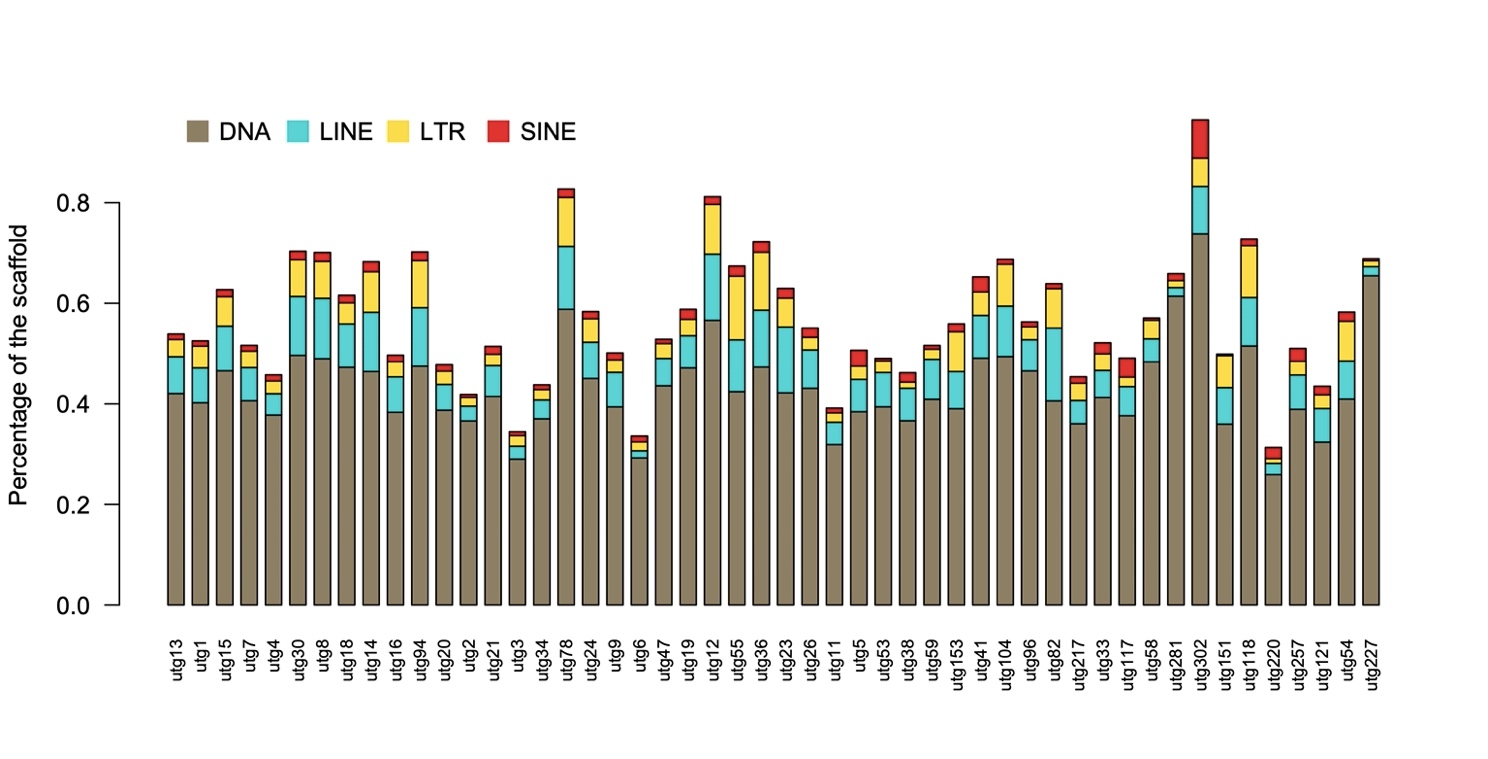


**Fig. S2** **Interspersed repeat landscape of the *Anagrus nilaparvatae* genome.** The top 50 longest scaffolds are presented, with decreasing size from left to right.

**
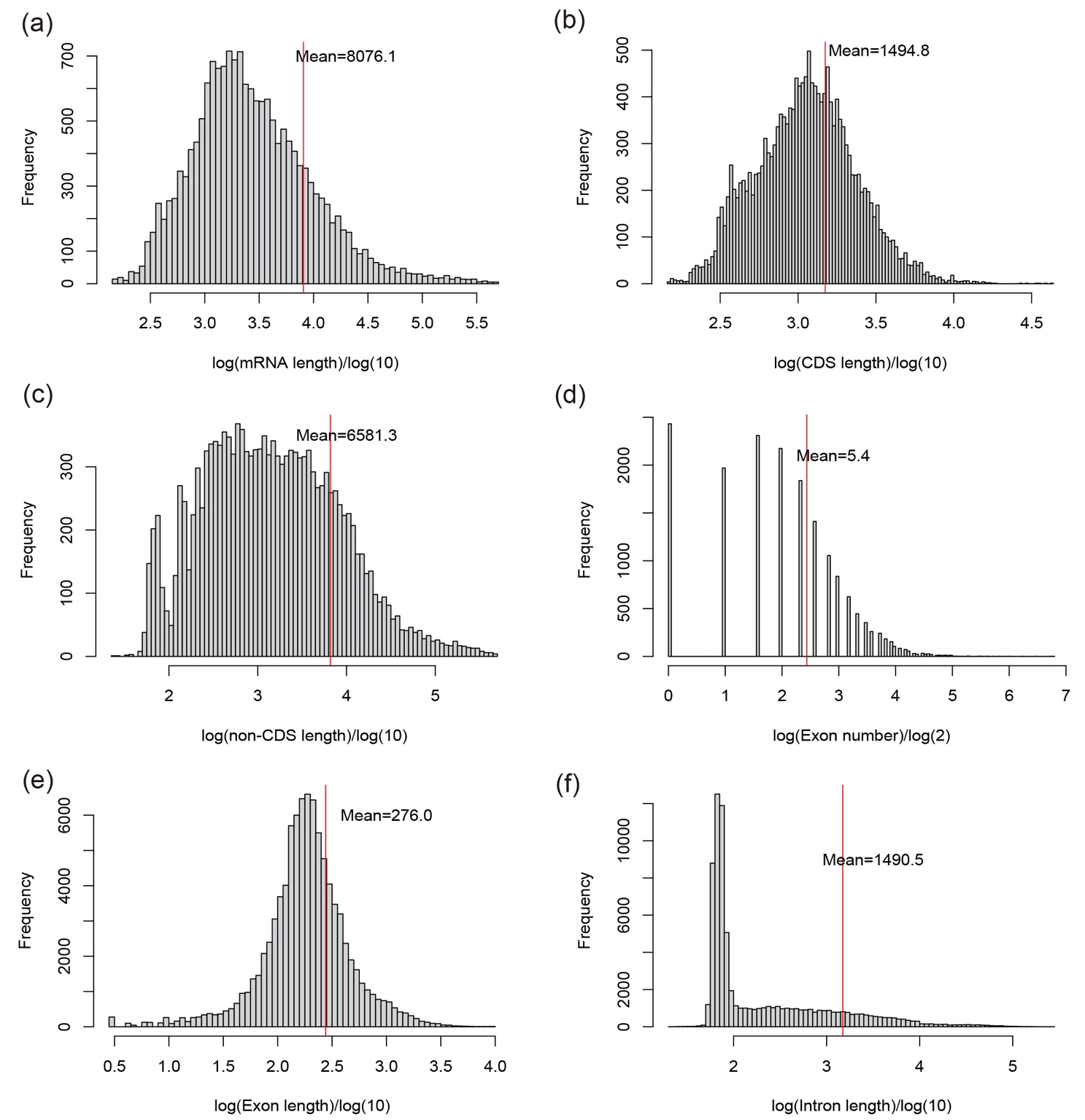
**

**Fig. S3 Distributions of the structural characters of the genes predicted in the *Anagrus* *nilaparvatae* genome.** (a) The distribution of mRNA length of all genes. (b) The distribution of Coding sequence (CDS) lengths of all genes. (c) The distribution of non-coding sequence (non-CDS) lengths of all genes. (d) The distribution of exon numbers in each gene. (e) The distribution of lengths of each exon. (f) The distribution of lengths of each intron.


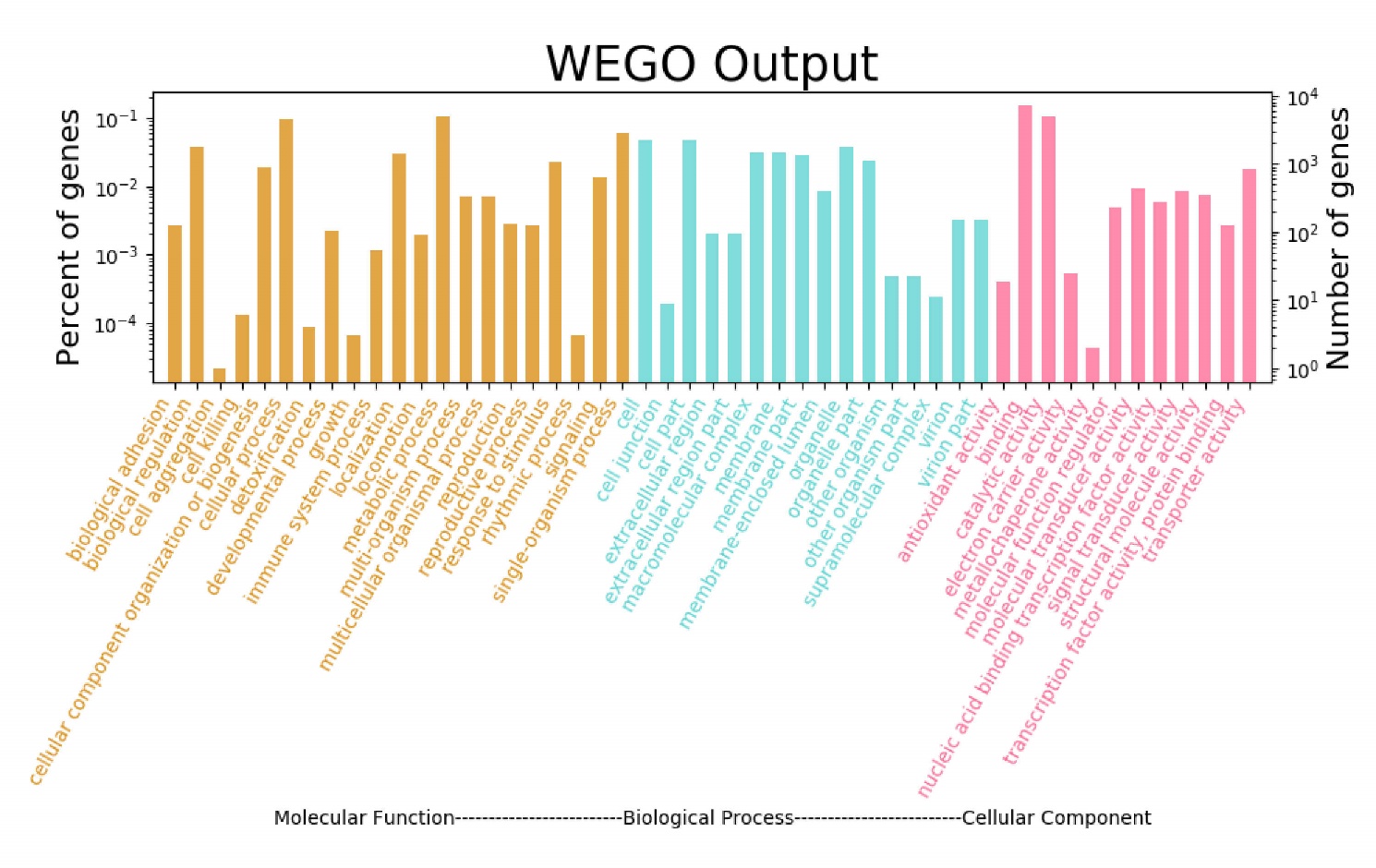


**Fig. S4 GO functional classification of the *Anagrus nilaparvatae* predicted genes.**


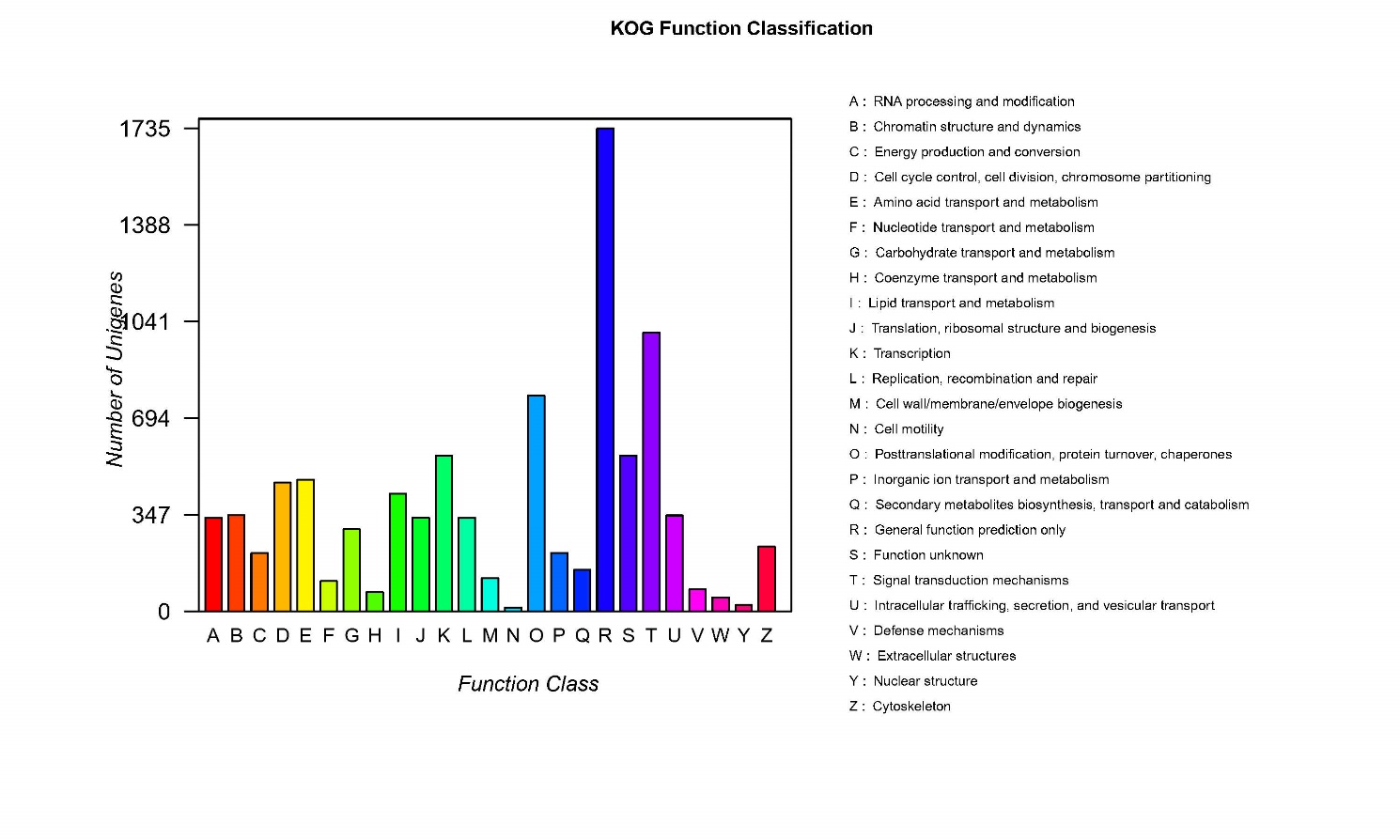


**Fig. S5** **KOG function classification of the predicted genes of *Anagrus nilaparvatae*.**


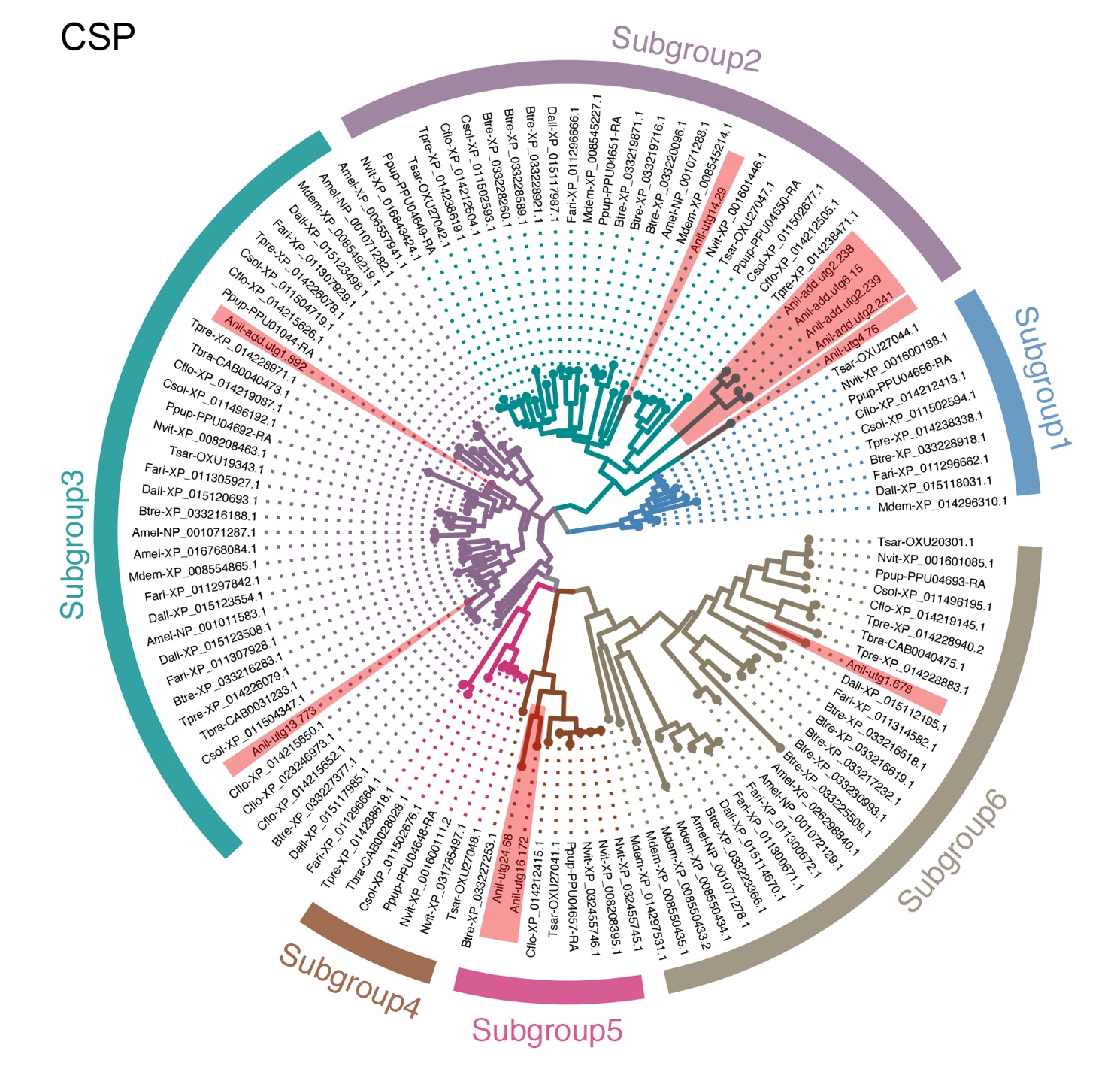


**Fig. S6** **Maximum-likelihood tree of CSPs of *Anagrus nilaparvatae* and other Hymenopteras.** All gene names are the abbreviation of the species name plus the gene serial number, the gene serial number could be found in NCBI (<https://www.ncbi.nlm.nih.gov/>) or InsectBase 2.0 (http://v2.insect-genome.com/); Anil, *Anagrus nilaparvatae*; Amel, *Apis mellifera*; Btre, *Belonocnema treatae*; Cflo, *Copidosoma floridanum*; Csol, *Ceratosolen solmsi*; Dall, *Diachasma alloeum*; Fari, *Fopius arisanus*; Mdem, *Microplitis demolitor*; Nvit, *Nasonia vitripennis*; Ppup, *Pteromalus puparum*; Tbra, *Trichogramma brassicae*; Tpre, *Trichogramma pretiosum*; Tsar, *Trichomalopsis sarcophagae*.


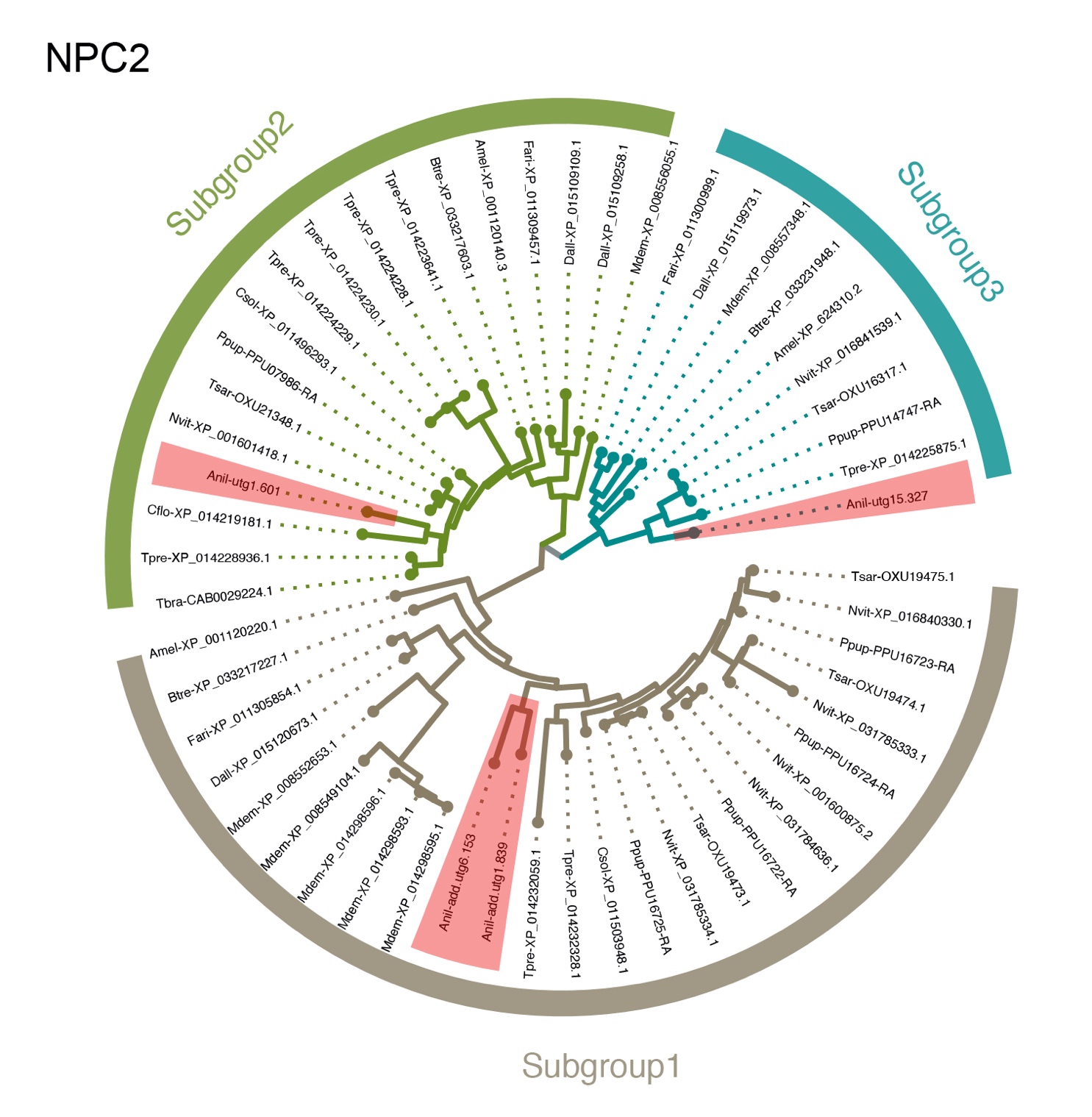


**Fig. S7** **Maximum-likelihood tree of NPC2s of *Anagrus nilaparvatae* and other Hymenopteras.** Same as Fig. S6.


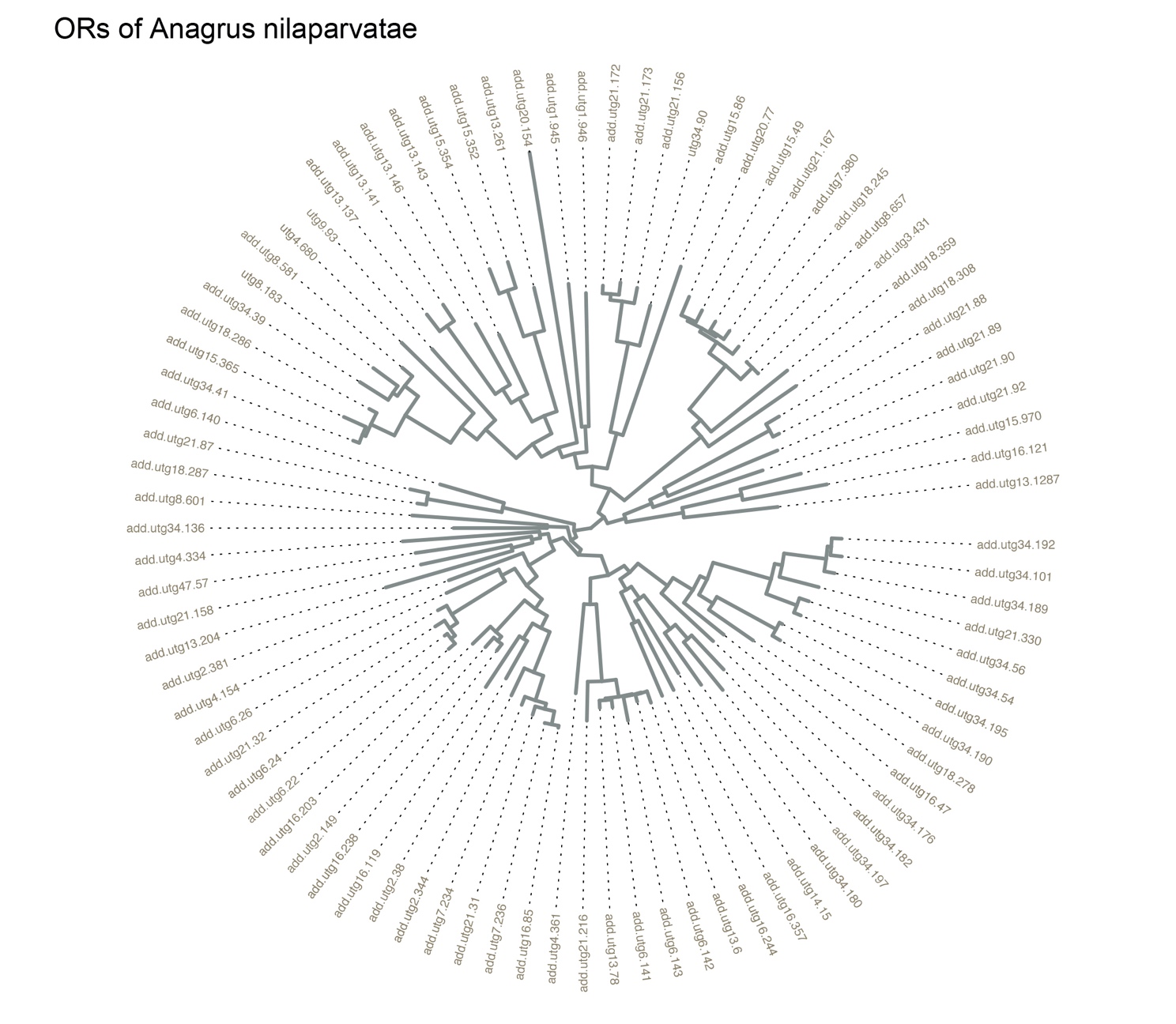


**Fig. S8 Maximum-likelihood tree of ORs of *Anagrus nilaparvatae* and other Hymenopteras.** Same as Fig. S6.


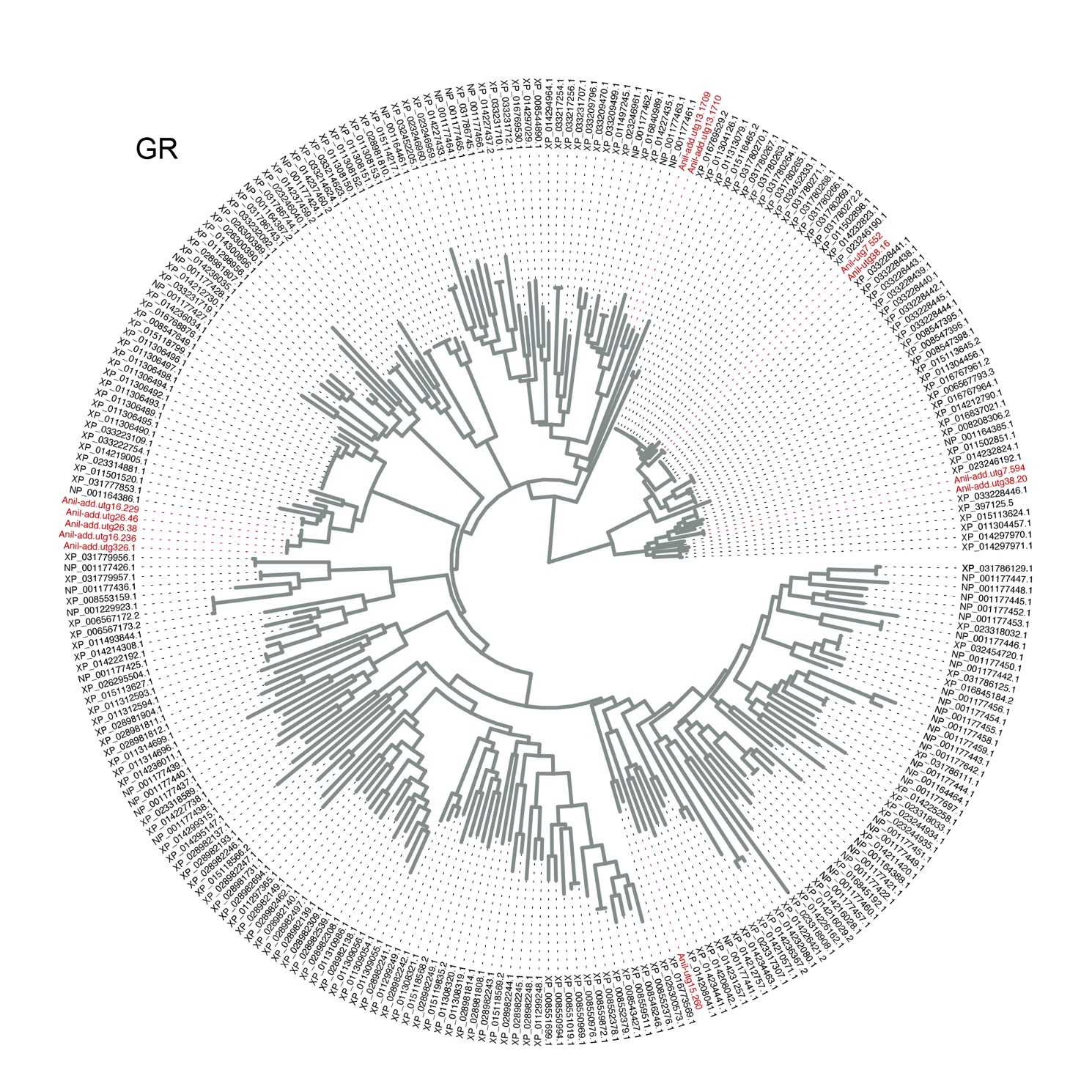


**Fig. S9** **Maximum-likelihood tree of GRs of *Anagrus nilaparvatae* and other Hymenopteras.** Same as Fig. S6.

**
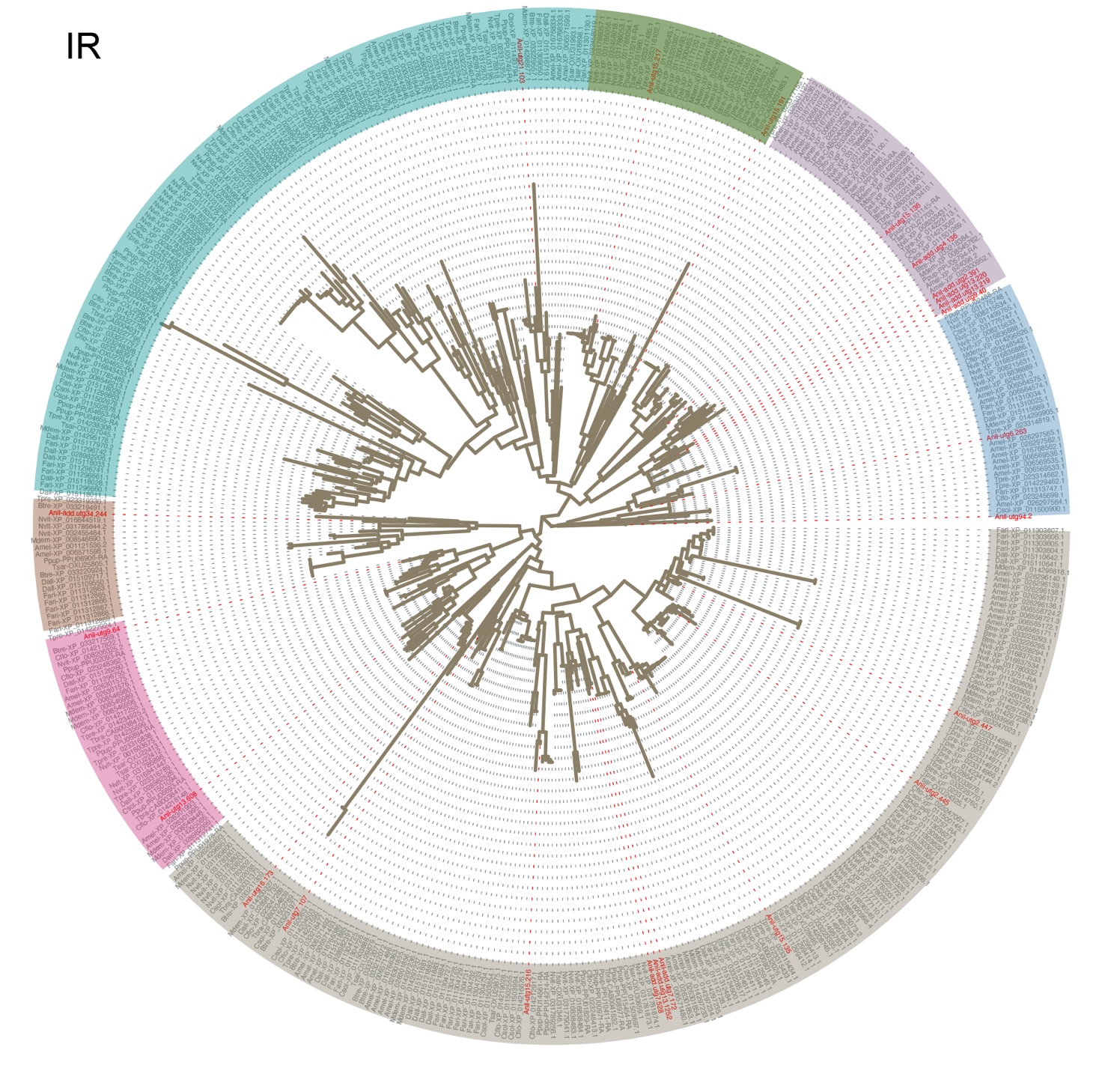
**

**Fig. S10** **Maximum-likelihood tree of IRs of *Anagrus nilaparvatae* and other Hymenopteras.** Same as Fig. S6.


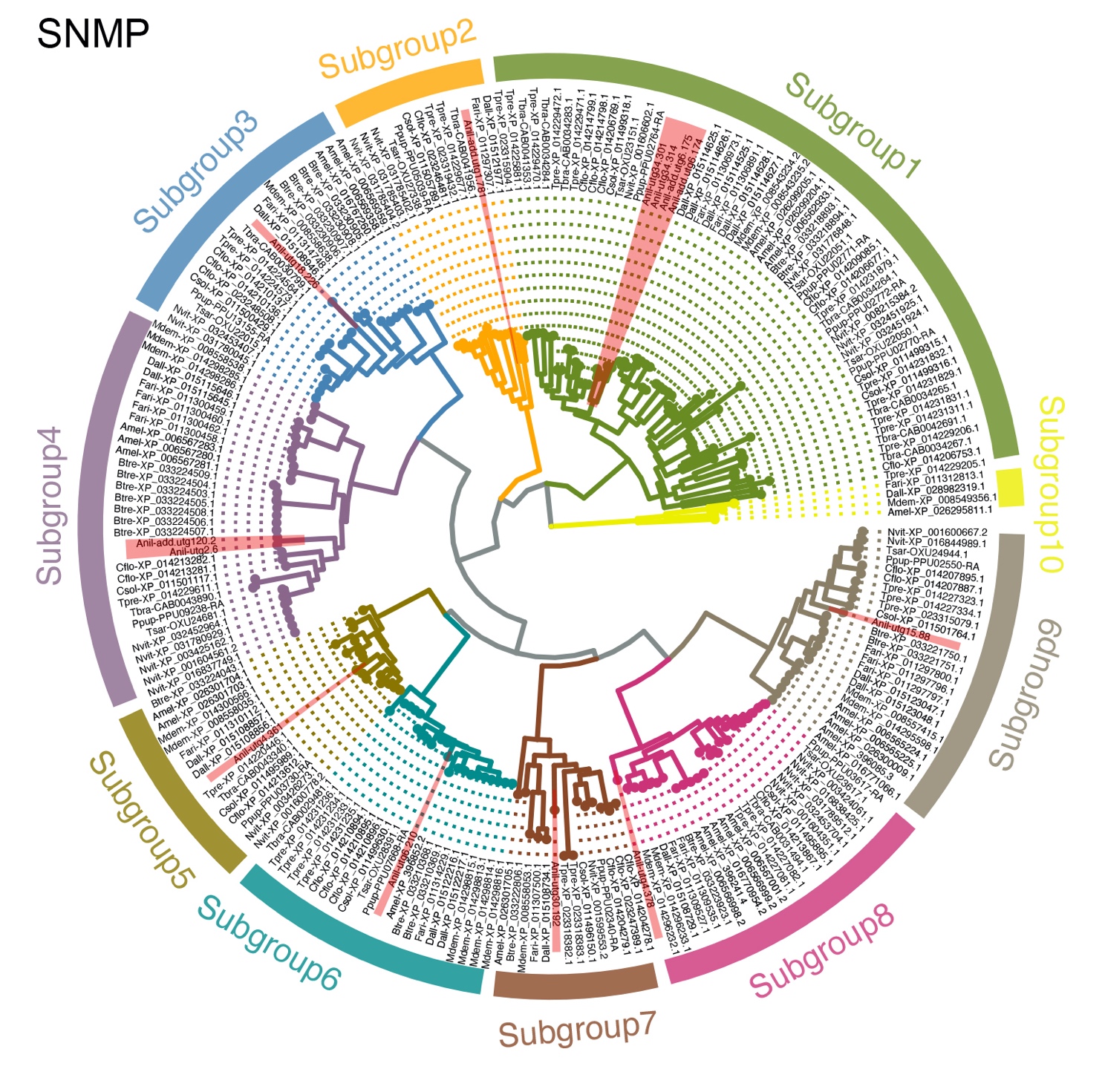


**Fig. S11** **Maximum-likelihood tree of SNMPs of *Anagrus nilaparvatae* and other Hymenopteras.** Same as Fig. S6.


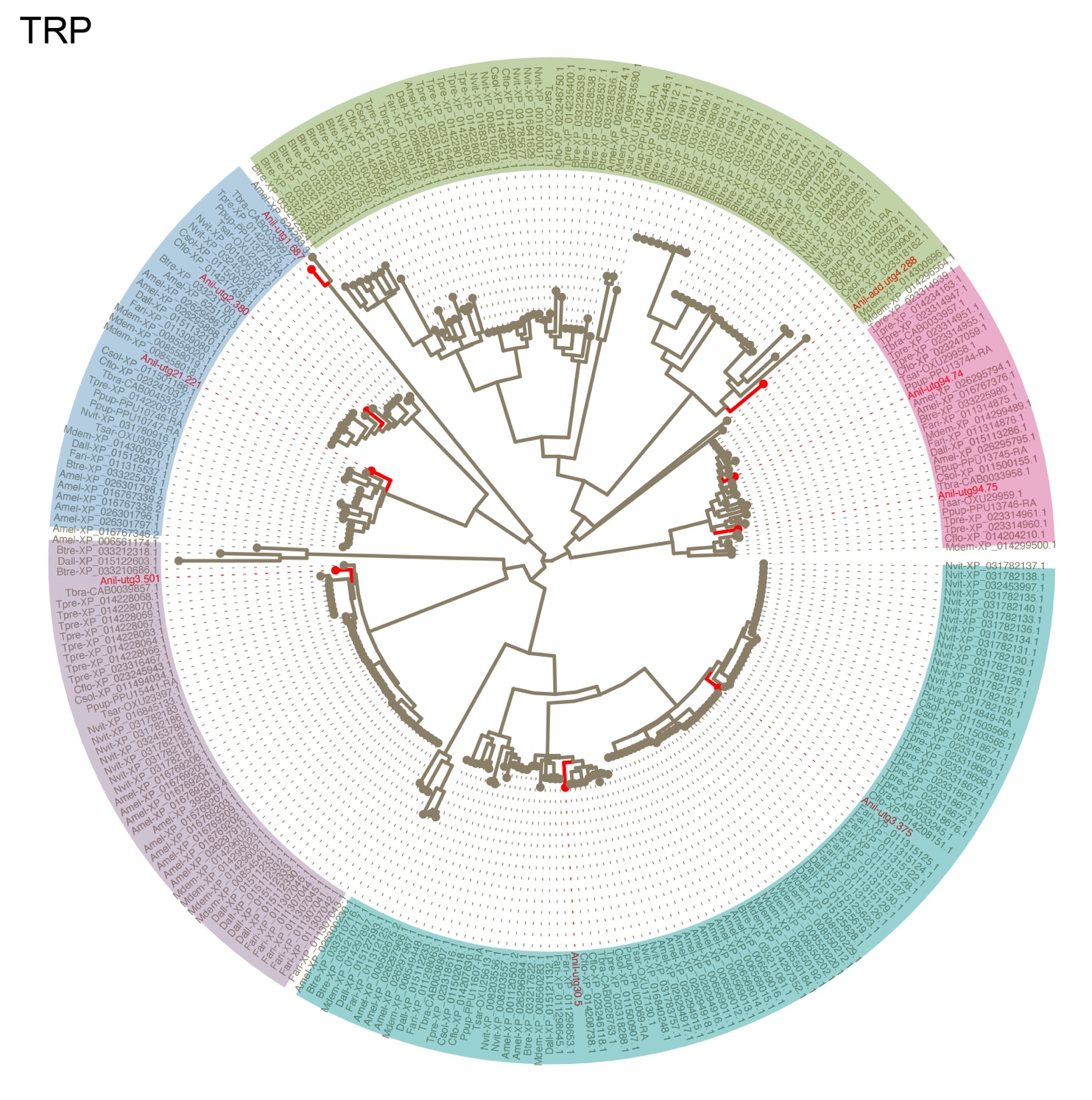


**Fig. S12** **Maximum-likelihood tree of TRPs of *Anagrus nilaparvatae* and other Hymenopteras.** Same as Fig. S6.
